# TiO_2_ Nanoparticles with Adjustable Phase Composition Prepared by an Inverse Microemulsion Method: Physicochemical Characterization and Photocatalytic Properties

**DOI:** 10.3390/nano14131130

**Published:** 2024-06-30

**Authors:** Bogna D. Napruszewska, Anna Walczyk, Dorota Duraczyńska, Joanna Kryściak-Czerwenka, Robert Karcz, Adam Gaweł, Paweł Nowak, Ewa M. Serwicka

**Affiliations:** 1 Jerzy Haber Institute of Catalysis and Surface Chemistry, Polish Academy of Sciences, Niezapominajek 8, 30-239 Krakow, Poland; ncnaprus@cyf-kr.edu.pl (B.D.N.); anna.walczyk@ikifp.edu.pl (A.W.); dorota.duraczynska@ikifp.edu.pl (D.D.); joanna.krysciak-czerwenka@ikifp.edu.pl (J.K.-C.); robert.karcz@ikifp.edu.pl (R.K.); ncnowak@cyf-kr.edu.pl (P.N.); 2 Faculty of Chemistry, Jagiellonian University Krakow, Gronostajowa 2, 30-387 Krakow, Poland; 3 Faculty of Geology, Geophysics and Environmental Protection, AGH University of Science and Technology, al. Mickiewicza 30, 30-059 Krakow, Poland; agawel@agh.edu.pl

**Keywords:** TiO_2_, nanoparticles, inverse microemulsion, anatase, rutile, brookite, photocatalysis, rhodamine B

## Abstract

Titania nanoparticles (NPs) find wide application in photocatalysis, photovoltaics, gas sensing, lithium batteries, etc. One of the most important synthetic challenges is maintaining control over the polymorph composition of the prepared nanomaterial. In the present work, TiO_2_ NPs corresponding to anatase, rutile, or an anatase/rutile/brookite mixture were obtained at 80 °C by an inverse microemulsion method in a ternary system of water/cetyltrimethylammonium bromide/1-hexanol in a weight ratio of 17:28:55. The only synthesis variables were the preparation of the aqueous component and the nature of the Ti precursor (Ti(IV) ethoxide, isopropoxide, butoxide, or chloride). The materials were characterized with X-ray diffraction, scanning/transmission electron microscopy, N_2_ adsorption–desorption isotherms, FTIR and Raman vibrational spectroscopies, and diffuse reflectance spectroscopy. The synthesis products differed significantly not only in phase composition, but also in crystallinity, textural properties, and adsorption properties towards water. All TiO_2_ NPs were active in the photocatalytic decomposition of rhodamine B, a model dye pollutant of wastewater streams. The mixed-phase anatase/rutile/brookite nanopowders obtained from alkoxy precursors showed the best photocatalytic performance, comparable to or better than the P25 reference. The exceptionally high photoactivity was attributed to the advantageous electronic effects known to accompany multiphase titania composition, namely high specific surface area and strong surface hydration. Among the single-phase materials, anatase samples showed better photoactivity than rutile ones, and this effect was associated, primarily, with the much higher specific surface area of anatase photocatalysts.

## 1. Introduction

Titanium dioxide nanomaterials are widely used in catalysis, photocatalysis, gas sensing, dye-sensitized solar cells, lithium batteries, etc. [1,2,3,4,5,6,7]. This broad range of applications is due to the combination of favorable characteristics exhibited by TiO_2_, in particular its high chemical stability, photoactivity, non-toxicity, environmental friendliness, and cost-effectiveness. These characteristics make titania attractive for the design of green chemistry materials and processes, especially those aimed at curbing energy deficits and environmental pollution. The design of TiO_2_ NPs for photocatalytic applications is currently the most dynamically developing research area [8,9,10]. 

The properties of TiO_2_ nanoparticles (NPs) depend on many factors, such as titania crystal structure, particle size and morphology, specific surface area, etc. As to their crystal structures, three TiO_2_ polymorphs exist in nature: rutile (tetragonal), anatase (tetragonal), and brookite (orthorhombic). In the case of macrocrystalline titania, rutile is the most stable form, while anatase and brookite are metastable and transform to rutile upon thermal treatment. However, the thermodynamic stability of TiO_2_ is particle-size dependent, and for crystallites of less than ca. 14 nm diameter, anatase becomes more stable than rutile [11]. In accordance with this finding, nanocrystalline TiO_2_ tends to crystallize as the anatase modification. 

Over the past few decades, a large variety of preparative approaches to synthesizing nanostructural TiO_2_ have been developed, based, among others, on sol-gel, hydrothermal, solvothermal, inverse water-in-oil microemulsion, or chemical vapor deposition routes. Their principles and outcomes are discussed in many reviews [1,3,12,13,14,15]. While all these methods have their advantages, synthesis via inverse microemulsion (also referred to as reverse microemulsion), appears particularly attractive [15,16,17,18,19]. This is due to the fact that inverse micelles are nanosized aqueous-phase droplets dispersed in a continuous organic phase, stabilized by the presence of surfactants at the water–oil interface. Formation of the desired product occurs in the aqueous micellar cores, the dimensions of which can be modified by the adjustment of the water/oil/surfactant ratio. For this reason, the method enables the preparation of monodispersed nanoparticles of controlled size and shape.

There are many papers describing the use of the inverse microemulsion method for the synthesis of nanosized TiO_2_ by the hydrolysis of various Ti precursors in aqueous micelle centers, e.g., [20,21,22,23,24,25,26,27,28,29,30,31,32,33,34,35,36,37]. Both inorganic (TiCl_4_ [27,28,34]) and, more frequently, organic compounds (Ti butoxide, Ti isopropoxide [20,21,22,23,24,25,26,29,30,31,32,33,35,36,37,38]) have been used as titanium sources, although their direct comparison in the same study is lacking. Typically, the oil phase has consisted of cycloalkanes (e.g., cyclohexane, cycloheptane) [20,21,22,23,24,25,26,27,30,32,34,36], while the stabilization of inverse micelles was usually ensured by the use of the non-ionic Triton-100X surfactant [20,21,22,24,26,31,32,34,36]. Many of the described preparative routes can be considered “microemulsion-mediated” because the as-received precipitates were usually amorphous and further autoclave thermal treatment or calcination was required to obtain specific crystal phases [20,21,22,24,25,27,28,29,30,31,32,33,35,36,38]. In general, thermal treatment initially led to the crystallization of anatase, while the formation of rutile required further increases of temperature. Unfortunately, high-temperature heating, whether during hydrothermal treatment or calcination, may degrade the properties of NPs due to coalescence and/or sintering. Examples of the retrieval of crystalline TiO_2_ NPs directly from the microemulsion environment without post-synthesis heat treatment are much less common [23,26,34,37].

To the best of our knowledge, this work describes for the first time the use of the same inverse microemulsion system to synthesize both phase-pure anatase and phase-pure rutile, as well as mixed-phase titania NPs under mild temperature conditions (80 °C), without the need for post-synthesis autoclaving or calcination. This paper also presents the first direct comparison of inverse microemulsion syntheses carried out with a series of different TiO_2_ precursors (TiCl_4_, Ti(IV) ethoxide, Ti(IV) isopropoxide, and Ti(IV) butoxide). The ternary system water/cetyltrimethylammonium bromide (CTABr)/1-hexanol with well-known phase diagram [39] was chosen for the preparation of microemulsions. 

The photocatalytic activity of the synthesized materials was evaluated in the photodegradation of rhodamine B, a compound modeling common dyes that pollute wastewater streams [40], and compared with the performance of P25, a commercial titania nanopowder, known for its exceptionally high activity in various photocatalytic reactions and widely used as a benchmark in TiO_2_-related studies [41].

## 2. Materials and Methods

### 2.1. Materials

TiCl_4_ (ReagentPlus®), Ti(IV) ethoxide (≥97%, referred to as TiEt), Ti(IV) isopropoxide (≥97%, referred to as TiProp), Ti(IV) butoxide (≥97%, referred to as TiBut), CTABr (≥98%), and 1-hexanol (for synthesis) were purchased from Sigma-Aldrich (Poznan, Poland); P25 TiO_2_ was manufactured by Evonik (Essen, Germany). All other chemicals used in syntheses were of p.a. purity provided by Chempur (Piekary Slaskie, Poland). Based on the water/CTABr/1-hexanol phase diagram [39], a 17:28:55 weight ratio of aqueous to surfactant to oil phase was chosen for the preparation of inverse microemulsions. 

The Ti source was introduced into the microemulsion either as the as-received compound or as a Ti-containing aqueous sol obtained by controlled hydrolysis of the appropriate Ti precursor. The TiCl-derived sol was prepared by the method established by Sterte [42]. Typically, 6.5 mL of 6 M HCl was added to 9 mL of titanium tetrachloride and diluted with distilled water to 100 mL. After stirring for up to 3 h at room temperature, a clear solution containing 3.7 wt.% of Ti was obtained. In the case of alkoxide precursors, 30 mL of 6 M HCl was mixed with the amount of alkoxide which, after dilution with distilled water to 100 mL, yielded a sol concentration of 3.7 wt.% of Ti (16.9 mL TiEt, 23.4 mL TiProp, and 27.0 mL TiBut). Clear solutions were obtained after short stirring at room temperature. 

Three variants of TiO_2_ NP synthesis, designated A, B, and C, were used, differing in the composition of the aqueous phase of the microemulsion and the manner of introducing the Ti source as follows:

- The first reverse microemulsion was prepared using distilled water as the aqueous phase. In a typical experiment, water, CTABr, and 1-hexanol were mixed together in a glass beaker in the weight ratio 17:28:55 and stirred magnetically until the mixture turned transparent, indicating the formation of an inverse microemulsion. Subsequently, the required Ti compound was added dropwise to the stirred microemulsion in the amount yielding 3.7 wt.% Ti in the aqueous phase. The liquid remained clear;
- The second reverse microemulsion was prepared using 6 M HCl solution as the aqueous phase. Otherwise, the synthetic procedure was identical to that of variant A. Also, here the obtained transparent inverse microemulsion remained clear after the addition of any of the Ti sources;
- The third reverse microemulsion was prepared using one of the prepared Ti sols as the aqueous component. In a typical experiment, aqueous Ti sol, CTABr, and 1-hexanol were mixed together in the weight ratio 17:28:55 and stirred magnetically until the mixture turned transparent.

**Figure 1:**
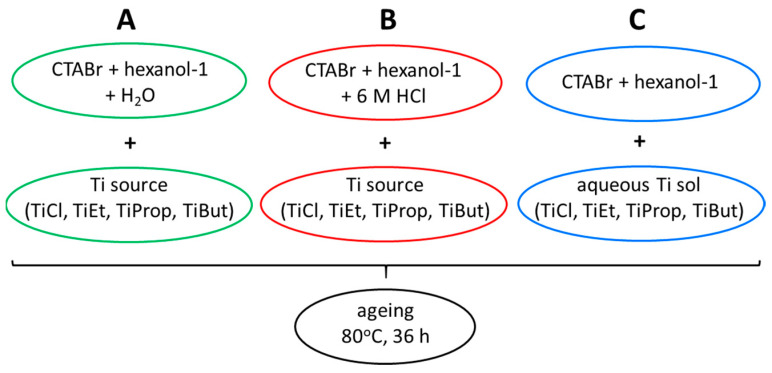
Schematic illustration of inverse microemulsion synthesis via route A, B, or C.

In order to promote the hydrolysis of Ti precursors, microemulsions obtained by any of the synthetic variants were heated to 80 °C, whereby the initially transparent inverse microemulsions turned into white suspensions, and aged at this temperature for 36 h in round bottom flasks with magnetic stirrers (500 rpm) using the thermostatic Radleys Carousel 6 parallel reaction station (Radleys, Shire Hill, Saffron Walden, Essex, UK). A schematic illustration of inverse microemulsion synthesis variants is shown in Figure 1. 

The precipitates obtained via route A, B, or C were recovered from the mother liquid and washed by centrifugation with 96 wt.% ethanol until free of bromide ions. After washing, all precipitates were dried in a drying box at 50 °C. The general signature of the prepared samples is “X-Precursor”, where X is A, B, or C, depending on the synthesis variant; the Ti precursor is indicated as TiEt, TiProp, TiBut, or TiCl. 

### 2.2. Methods

Powder X-ray diffraction (XRD) patterns were recorded using an X’Pert PRO MPD diffractometer (PANalytical, Almelo, The Netherlands) with CuKα radiation (40 kV, 30 mA), a flat graphite monochromator with a diffracted beam, and a step size of 0.0334°. TiO_2_ phase identification was based on comparison with the JCPDS reference patterns (01-075-2546 for anatase, 04-005-4859 for rutile, and 01-075-2549 for brookite). In mixed-phase titania samples, contributions of various phases were evaluated by the method of Zhang and Banfield [11] from the integrated intensities of the anatase (101), brookite (121), and rutile (110) XRD peaks. The crystallite sizes of the different TiO_2_ phases (i.e., the sizes of coherently scattering domains) were calculated using Scherrer’s formula, based on the FWHM (full width at half maximum) of the same reflections, taking into account instrumental broadening. Due to the overlap of the (101) anatase reflex with the (120) and (111) brookite reflexes, a deconvolution was performed in the scanning 2θ range 22–34^o^, using lorentzian functions and Fityk software 0.9.8 [43] to separate the peaks. The fitting procedure assumed that the broadening of the brookite reflections (120), (111), and (121) were the same, and the ratio of their intensities was constant and corresponded to that in the reference pattern JCPDS 01-075-2549.

The scanning/transmission electron microscopy (SEM/TEM) study of the materials was carried out with aid of a JEOL JSM-7500F Field Emission Scanning Electron Microscope (JEOL, Tokyo, Japan). SEM/TEM images were recorded for the uncoated samples deposited as suspensions on 200 mesh copper grids covered with carbon support film. 

Transmission Fourier transform infrared (FTIR) spectra were recorded using a Nicolet 6700 (Thermo Scientific, Madison, WI, USA) spectrometer in the 4000–400 cm^−1^ range, at a spectral resolution of 2 cm^−1^ for samples prepared as KBr discs. 

Raman spectra were collected with a DXR Raman microscope (Thermo Scientific, Waltham, MA, USA) using a 532 nm excitation laser wavelength, power level of 6 mW, and spectral resolution of 2 cm^−1^. 

N_2_ adsorption/desorption at −196 °C was measured with an AUTOSORB 1 (Quantachrome, Boynton Beach, FL, USA) instrument. The samples were outgassed at 150 °C for 2 h. Brunauer–Emmett–Teller (BET) formalism was used for the calculation of specific surface areas (S_BET_). The total pore volume (V_tot_) was determined from the amount of N_2_ adsorbed at p/p_0_ = 0.996. Pore size distribution (PSD) profiles were determined from the adsorption branch using the non-local density functional theory (NL DFT) method. The mean pore diameter (D_av_) was calculated with the D_av_ = 4V_tot_/S_BET_ Gurvitch formula. 

UV–visible diffuse reflectance spectra (DRS) of the pelletized powder samples were recorded from 250 nm to 900 nm using a Perkin Elmer UV-Vis spectrometer model Lambda 35 (PerkinElmer, Waltham, MA, USA) equipped with a diffuse reflectance accessory. The band gap energies, Eg, were evaluated by converting the spectra to Kubelka–Munk functions, derived from the reflectance spectrum using the equation F(R) = (1 − R)^2^/(2R), where R is the reflectance, and graphical extrapolation of the linear part of the F(R) plot to the energy axis [44]. 

The photocatalytic activity of the synthesized TiO_2_ samples was evaluated in the decomposition of rhodamine B (RhB) (≥95%, Sigma-Aldrich, Poznan, Poland) under UV light. The reaction was carried out in a beaker made of Pyrex glass transparent to the UV radiation used in the experiments, which was then placed on a magnetic stirrer and illuminated from the top by a 365 nm light-emitting diode (LED) 50 W lamp (EcoEnergy, Słupsk, Poland). The whole setup was surrounded by a light-reflecting aluminum housing. To avoid raising the temperature inside the housing, the reactor was cooled by an electric fan. In a typical experiment, 0.02 g photocatalyst was placed in 50 mL 10^−5^ M RhB aqueous solution and stirred for 1 h in the dark to ensure an adsorption–desorption equilibrium before illumination. A 3 mL sample was taken at 1 h intervals, centrifuged, and the concentration of RhB was determined by measuring light absorption at 555 nm (λ_max_ for RhB) using a Lambda 35 Perkin Elmer UV-Vis spectrometer (PerkinElmer, Waltham, MA, USA). The photodegradation of RhB is presented in terms of C/C_0_ vs. illumination time, where C is the absorbance of the RhB solution measured at 1 h intervals, and C_0_ is the absorbance of the RhB solution before irradiation.

## 3. Results and discussion

### 3.1. X-ray Diffraction Analysis

**Figure 2:**
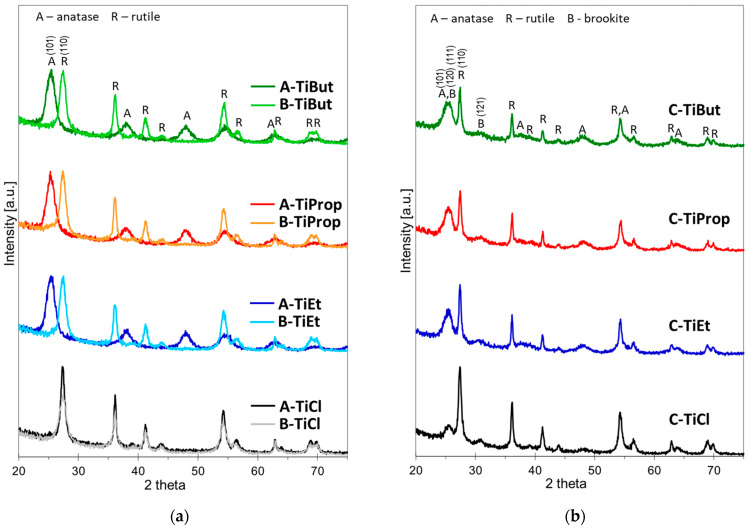
XRD patterns of TiO_2_ nanoparticles obtained from different Ti sources by three variants of synthesis: (**a**) direct comparison of method A and B; (**b**) method C.

All materials prepared according to the scheme presented in Figure 1 displayed XRD patterns characteristic of different crystalline titania phases (Figure 2). Figure 2a enables the comparison of the diffractograms of samples obtained by methods A and B, i.e., in processes in which Ti precursors were added directly to pre-formed microemulsions that contained pure water or 6 M HCl solution as the aqueous phase, respectively. It is clear that, for alkoxide precursors, the composition of the aqueous phase plays a decisive role in determining the nature of the titanium phase that precipitates in the micellar cores. Thus, in the A series samples, where water is used as the aqueous phase, Ti ethoxide, isopropoxide, and butoxide yielded anatase as the only TiO_2_ modification identified by XRD. The size of anatase crystallites, estimated using the Scherrer equation, was similar for all Ti alkoxide precursors and ranged from 6.2 to 6.5 nm (Table 1).

In contrast, in series B, the environment of 6 M HCl led to the formation of rutile from all alkoxide sources. The size of rutile crystallites ranged from 8.1 to 8.7 (Table 1). 

In the case of the TiCl_4_ precursor, rutile formation was observed regardless of whether the aqueous phase was pure water or 6 M HCl. 

A different situation arises when the aqueous phase used for the formation of microemulsions contains Ti precursor, as in series C (Figure 2b). Here, as a rule, multiphase titania precipitates are formed, indicating the coexistence of anatase, rutile, and/or brookite, of which rutile shows enhanced crystallinity (18.2–24.8 nm) with respect to anatase (5.1–5.9 nm) and brookite (7.6–9.3 nm). Quantitative estimates of the contribution of different TiO_2_ phases and the corresponding crystal sizes are given in Table 1. 

In an attempt to explain the observed variation in phase composition depending on the method of synthesis, it should be recalled that the formation of a particular TiO_2_ polymorph strongly depends on hydrolysis conditions, especially acidity. It has been shown that low acidity, which accelerates hydrolysis, favors the crystallization of metastable phases, i.e., anatase and brookite, while in the conditions of high acidity and slow condensation rate, the preferential formation of rutile is observed [45]. In method A, the alkoxide precursor is dissolved in purely aqueous micellar cores, in method B the micellar cores contain 6 M HCl, and in method C the concentration of HCl in Ti sol forming the micellar cores is about 2 M. Accordingly, method A yields anatase, method B gives rutile, while method C, with intermediate acidity, produces mixed-phase materials. The TiCl_4_ precursor differs fundamentally from alkoxides in that in an aqueous environment it becomes a source of additional HCl, thereby increasing acidity and promoting rutile formation.

### 3.2. SEM/TEM Analysis

**Figure 3:**
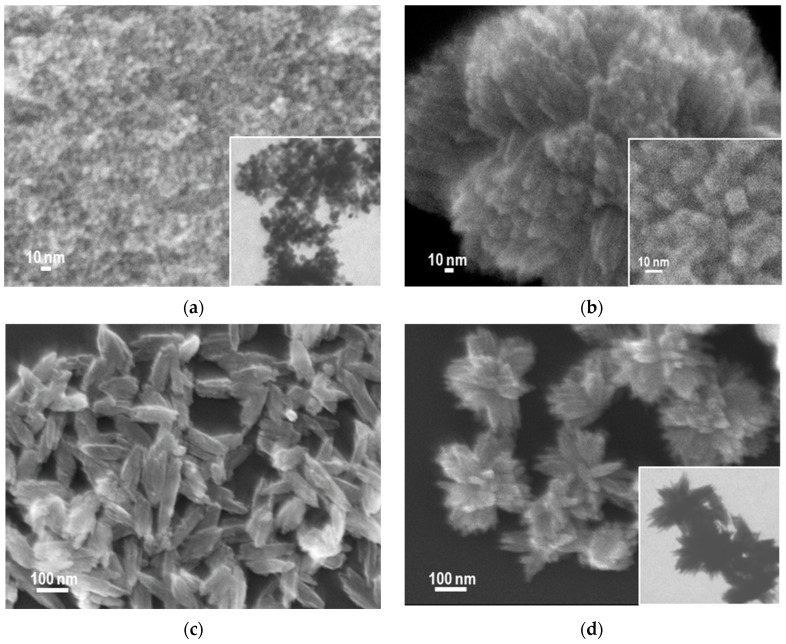

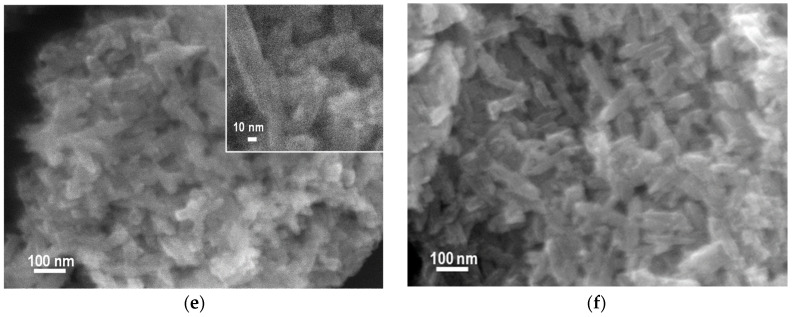
Electron microscopy images of titania obtained by means of inverse microemulsion: (**a**) SEM of A-TiEt (insert: TEM image of the same magnification); (**b**) B-TiProp (insert: a blown-up SEM image showing square tops of rod-like particles); (**c**) SEM of A-TiCl; (**d**) SEM of B-TiCl (insert: TEM image of the same magnification); (**e**) SEM of C-TiBut (insert: a blown-up SEM image of non-uniform small particles covering nanorods); (**f**) SEM of C-TiCl.

SEM and, in selected cases also TEM micrographs, of TiO_2_ nanopowders obtained by means of inverse microemulsion from different precursors with the use of different methods are gathered in Figure 3. All titania samples prepared from Ti alkoxides by method A, and characterized by anatase structure, appear to consist of rounded, fairly uniform particles of ca. 10 nm diameter. Figure 3a shows, as an example, a SEM micrograph with an insert of TEM image of an A-TiEt sample. Switching to method B resulted in a dramatic change of morphology of titania samples derived from Ti alkoxides. A common feature of the rutile samples obtained in this way is the formation of spherically packed bundles of nanorods, with spheroid diameters on the order of several hundred nm, as illustrated in Figure 3b with an SEM image of the B-TiProp sample. Clearly visible square tops of individual nanorods (insert in Figure 3b) indicate that the rutile NPs have tetragonal habit. TiO_2_ NPs obtained from the Ti tetrachloride precursor by methods A and B show yet another morphology. Sample A-TiCl is composed of spindle-shaped grains of length around 100 nm (Figure 3c). In sample B-TiCl, the elongated particles are smaller and form flower-like clusters. The TEM image (insert) shows that the elongated grains are agglomerates of thinner, needle-like structural subunits (Figure 3d). In the case of samples prepared by method C, irrespective of the nature of the Ti precursor, similar morphological features are observed. All precipitates are a mixture of rod-like grains several tens of nm long and finer irregular particles. SEM of C-TiBut (Figure 3e) exemplifies the typical morphology of alkoxide-derived materials. At a higher magnification, as in the insert to Figure 3e, the small particles covering long grains appear non-uniform. The morphology of sample C-TiCl, shown in the SEM image presented in Figure 3f, is very similar, although small particles appear less abundant. 

### 3.3. Textural Properties

**Figure 4:**
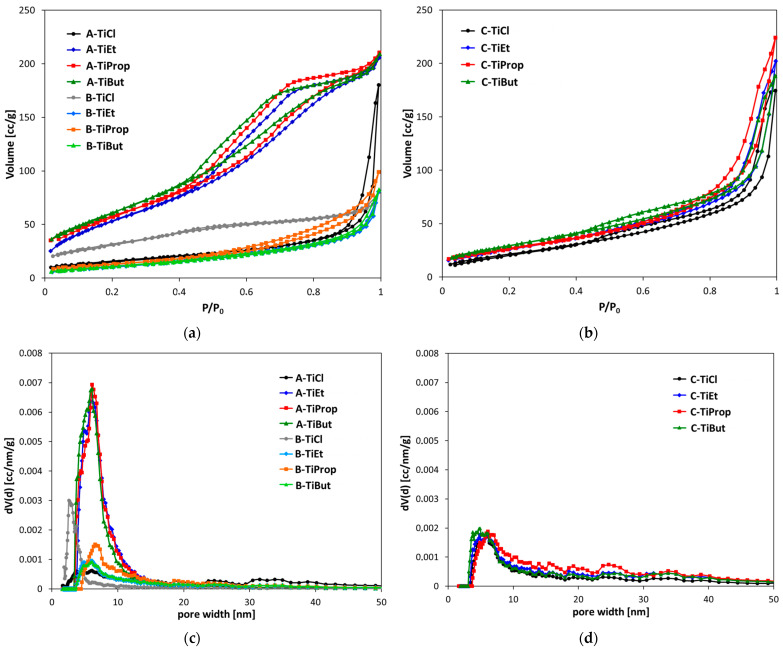
N_2_ adsorption/desorption of isotherms at –196 °C of: (**a**) TiO_2_ NPs prepared by methods A and B; (**b**) TiO_2_ NPs prepared by method C. Differential pore size distributions of: (**c**) TiO_2_ NPs prepared by method A and B; (**d**) TiO_2_ NPs prepared by method C.

The N_2_ adsorption/desorption isotherms of TiO_2_ NPs are shown in Figure 4a,b, the former showing the results for samples obtained by method A and B, and the latter for materials synthesized by method C. The corresponding pore size distribution (PSD) curves are shown in Figure 4c,d. Textural parameters are listed in Table 1. The graphs obtained for samples prepared by a particular method vary considerably, and, in the case of methods A and B, depend also on the nature of the Ti precursor. For samples synthesized with the use of alkoxide Ti precursors, the isotherms of materials obtained by a given synthesis variant are very similar to each other. On the other hand, the sets of isotherms associated with a particular method (A, B, or C) differ, which is obviously related to the different morphology of the powders tested. Thus, isotherms of A-TiEt, A-TiProp, and A-TiBut, which, according to SEM/TEM analysis, are composed of rounded particles of ca. 10 nm diameter (Figure 3a), can be all classified as type II, following the IUPAC classification [46]. A characteristic feature of type II isotherms is a significant gas uptake at a medium and higher relative pressure range, appearing to increase without limit when p/p_0_ = 1. The observed hysteresis loops are complex, resembling the H2 type in the lower p/p_0_, and the H3 type at higher p/p_0_ values. The H3 type contribution is manifested by the lack of plateau in the high p/p_0_ value, and suggests that the pore network contains macropores which are not completely filled with pore condensate. Simultaneously, the lack of steep closure at p/p_0_ ~ 0.4 is a feature characteristic of the H2 type hysteresis loop, indicative of the pore blocking caused by the ink-bottle-like pores. Therefore, the porosity of TiO_2_ nanopowders obtained by method A is considered to stem chiefly from the interparticle voids within the aggregates of rounded titania NPs. Due to the strong overlapping of isotherms, the samples are characterized by similar textural parameters (Table 1). They display very high specific surface areas (over 200 m^2^/g) and total pore volumes around 0.3 cm^3^/g. NL DFT pore size distribution (PSD) curves of the samples practically overlap (Figure 4c). The dominant PSD maximum is in all cases around 6 nm and roughly equals the average pore diameter, in accordance with the relatively narrow PSD. 

The isotherms recorded for TiO_2_ NPs obtained from alkoxide precursors by method B differ very significantly from those synthesized by method A. Although they also may be described as type II with H2/H3 hysteresis loops, they lie much lower and the hysteresis loops are much narrower, indicating a lower adsorption capacity and lower macro/mesoporosity of the tested materials. As a result, the specific surface areas of the samples are significantly lower (around 40–50 m^2^/g) and so are the total pore volumes (around 0.13–0.15 cm^3^/g) (Table 1). This is consistent with the change in the morphology of TiO_2_ NPs, which in samples prepared from alkoxides by method B form spheroidal agglomerates with diameters measuring several hundred nm, composed of well-developed rutile nanorods (Figure 3b). Noteworthy, the dominant PSD peak maxima are similar to those found in samples prepared by method A (5.9–6.6 nm) but the average pore diameters are about twice as large. The latter effect is due to the strong asymmetry of the PSD curves, tailing off in the direction of higher pore width (Figure 4c).

The textural properties of materials obtained from alkoxides by method C, which morphologically are mixtures of larger nanorods and finer particles (Figure 3e), are in some respects intermediate between those exhibited by type A and type B samples, and in other respects closer to one of these groups. As in previous cases, the isotherms are of type II. They display well-defined narrow H3 hysteresis loops. At low p/p_0_, the isotherms of C-TiEt, C-TiProp, and C-TiBut lie between the ones recorded for samples obtained by methods A and B. This is reflected in the intermediate values of specific surface areas (99–112 m^2^/g) (Table 1). On the other hand, the strong upward swing at higher relative pressures, pointing to the presence of larger meso/macropores, accounts for the high overall adsorption capacity. As a consequence, the total pore volumes in series C are close to those observed for materials prepared by method A (Table 1). The dominant PSD peak maxima are in the range of 4.9 to 6.1 nm, but, as with samples synthesized by method B, the PSD curves are skewed toward the larger pore width (Figure 4d), which results in average pore diameters being much larger (10.0–14.0 nm) than the dominant pore sizes (Table 1). 

The isotherms obtained for TiO_2_ NPs prepared from the Ti tetrachloride precursor by methods A and B differ from the sets of isotherms for analogous materials derived from alkoxides, and also differ significantly between each other (Figure 4a). This is in obvious connection with their unique morphology. Thus, the initial part of the A-TiCl isotherm, which can be classified as type II with H2/H3 type hysteresis, lies much lower than the rest of isotherms of A-samples prepared from alkoxides. As a result, the material is characterized by a much lower specific surface than the rest of series A. However, it displays a strong upward turn at high relative pressures, due to the meso/macroporosity resulting from interparticle voids between the spindle-like grains identified by SEM (Figure 3c), so that the total sorption capacity of A-TiCl is high. The high contribution of large pores is reflected in the average pore size being more than three times that of the value of the dominant PSD maximum (19.8 vs. 6.1 nm) (Figure 4c, Table 1). The initial part of the B-TiCl isotherm lies higher than that of A-TiCl, resulting in a higher specific surface area, but the maximum gas uptake is low, and the isotherm has no hysteresis (Figure 4a). The PSD maximum occurs at 2.9 nm, which is close to the average pore size of 3.4 nm (Figure 4c, Table 1) and shows that the porosity is dominated by small mesopores, most likely originating from the packing of needle-like particles forming flower-like structures visible in SEM images (Figure 3d). TiO_2_ nanoparticles obtained from TiCl_4_ by the C method show yet other textural properties. In the low relative pressure range, the C-TiCl isotherm (Figure 4b) is positioned between the isotherms of A-TiCl and B-TiCl (Figure 4a), which results in the intermediate specific surface area, while the sharp upward sway of the isotherm at high relative pressures leads to the total pore volume similar as in the case of A-TiCl (Table 1). The isotherm of C-TiCl resembles closely the isotherms of TiO_2_ NPs derived by method C from alkoxides, as expected for materials of similar grain morphology (Figure 3e,f). The PSD curve is also similar to those obtained for samples prepared from alkoxides, and shows a maximum around 4.9 nm, but the contribution of larger pores, indicated by PSD curve asymmetry, results in the APD of 13 nm. 

### 3.4. Vibrational Spectroscopy

#### 3.4.1. FTIR Spectroscopy

**Figure 5:**
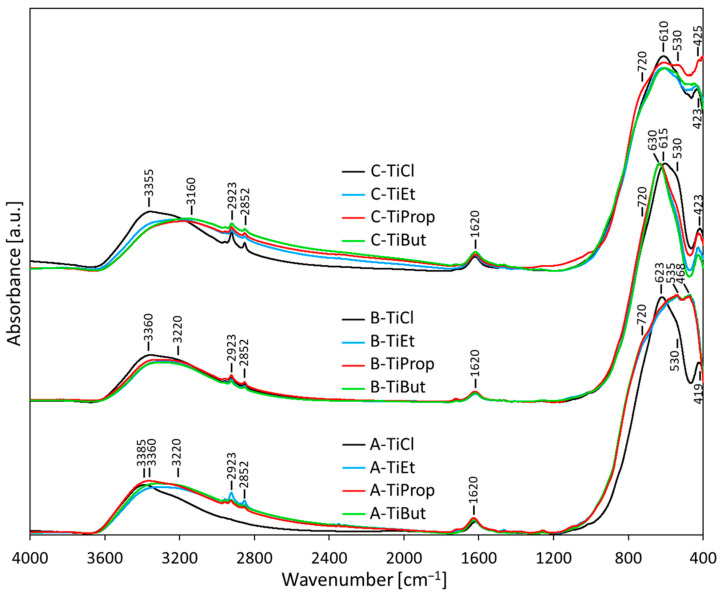
FTIR spectra of TiO_2_ NPs synthesized from different precursors by inverse microemulsion via route A, B, or C and dried at 50 °C.

Figure 5 shows the IR spectra of all prepared titania nanopowders, grouped by synthesis variant. In general, the sets of spectra recorded for TiO_2_ NPs obtained from alkoxides by a given synthesis method are very similar to each other, but different synthesis variants lead to different spectral features. For each preparative procedure, the spectra of TiO_2_ NPs obtained from TiCl_4_ are markedly different from those exhibited by samples derived from alkoxides. Above 3000 cm^−1^, where OH group stretching modes occur, intense, broad bands are observed with maxima between 3385 and 3160 cm^−1^. They originate from the stretching vibrations of hydrogen-bonded hydroxyl groups belonging to water molecules adsorbed at the surface. The lower the wave number of the maximum, the stronger the corresponding hydrogen bond [37]. It is apparent that water adsorbed at the surface of samples obtained from alkoxides by method C contains the highest share of strongly hydrogen-bonded hydroxyls, as indicated by the elevated broad maximum at 3160 cm^−1^. FTIR absorption profiles of OH stretching vibrations in samples obtained from TiCl_4_ are dominated by the maxima around 3355–3385 cm^−1^, pointing to the prevalent presence of weakly hydrogen-bonded hydroxyl groups. In addition, in all samples the bending mode of adsorbed water gives a maximum at 1620 cm^−1^. The sharp low-intensity maxima at 2923 and 2852 cm^−1^ are associated, respectively, with antisymmetric and asymmetric vibrations of C-H bonds in adsorbed organic species stemming from the atmosphere or from organic reactants used in syntheses. 

Below 1000 cm^−1^, in the spectral range revealing skeletal modes, intense broad bands with multiple maxima appear in all spectra, caused by vibrations of Ti-O bonds within the TiO_2_ lattice. All spectra of TiO_2_ NPs with an anatase structure are practically identical (A-TiEt, A-TiProp, and A-TiBut in Figure 5). In view of the XRD data, the broad band with maxima at 438 and 535 cm^−1^ and a shoulder at 720 cm^−1^ are attributed to vibrations within the anatase structure, which is supported by data from the literature [47,48,49,50,51,52]. The spectra of samples with an exclusively rutile structure (A-TiCl, B-TiEt, B-TiProp, B-TiBut, and B-TiCl in Figure 5) differ from those of anatase. They are dominated by a maximum around 615–630 cm^−1^, with shoulders at ca. 720 and 530 cm^−1^, and a clearly resolved relatively narrow maximum at ca. 419–423 cm^−1^, all of which are consistent with rutile formation [47,48,49,50,51,52].

The set of samples prepared by method C, with multiphase anatase/brookite/rutile composition identified by XRD, display FTIR spectra which agree with these findings. Thus, the strong maximum around 610 cm^−1^ and the narrow maximum around 423–425 cm^−1^ originate from the rutile component. The pronounced shoulder at 720 cm^−1^ and enhanced absorbance level between the 610 and the 425 cm^−1^ maxima are consistent with the presence of anatase and brookite (for which the skeletal spectra are very similar to that of anatase [52]). 

#### 3.4.2. Raman Spectroscopy

Raman spectroscopy is an important tool in the study of TiO_2_ because at low wavenumbers, where oxide lattice vibrations occur, different titanium polymorphs give clearly distinguishable spectra. Moreover, Raman spectroscopy is more sensitive to short-range order than XRD and can reveal trace phases that might otherwise go undetected by XRD [53]. For this reason, it is particularly suitable for corroborating XRD data obtained for nanocrystalline and/or poorly ordered solids. 

**Figure 6:**
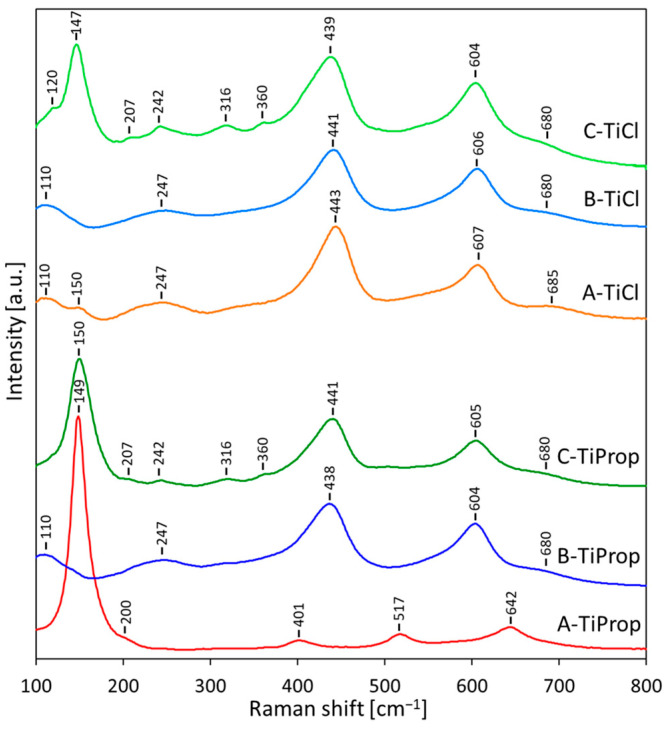
Raman spectra of TiO_2_ NPs synthesized from different precursors by inverse microemulsion via route A, B, or C and dried at 50 °C.

Figure 6 allows a comparison of the Raman spectra of TiO_2_ NPs obtained in synthesis variants A, B, or C using Ti alkoxide or Ti tetrachloride as a precursor. Samples prepared from Ti isopropoxide were taken as an example of alkoxide-derived materials. 

The spectrum recorded for A-TiProp, which according to XRD represents anatase, shows four distinct bands, located at 149, 401, 517, 642 cm^−1^, and a shoulder at 200 cm^−1^. All these maxima are characteristic of anatase, which has six Raman active modes. In well-crystalline oxide, they appear at 144, 197, 399, 513, 519 (superimposed with the 513 cm^−1^ one), and 639 cm^−1^ [54]. The observed blue shifts in the Raman spectrum of A-TiProp are related to the nanosize nature of titania particles [55,56,57]. 

The band maxima in the spectrum of the B-TiProp sample, which, according to XRD is rutile, appear at 110, 247, 438, 604, and 680 (shoulder) cm^−1^. Their positions differ significantly from the conventional spectrum of well-crystalline rutile, which displays four first order Raman active bands at 143, 230-240 (broad band, due to second-order scattering and disorder effects), 447, 612, and 826 cm^−1^ [54]. This is due to the fact that the Raman spectrum of rutile is strongly size-dependent [58,59], and in spectra of nanocrystalline rutile the appearance of new bands (around 110 and 680 cm^−1^) and shifts of band positions are observed. Ocana et al. [58] postulated that the new bands originate from the surface layers of nanocrystals and result from the relaxation of selection rules. The spectrum of B-TiProp is therefore consistent with the nanocrystalline nature of the rutile particles that make up this sample. 

Sample C-TiProp, which, according to XRD, is a mixture of rutile, anatase, and brookite, displays Raman spectrum in which next to the features attributable to rutile and anatase, several additional low intensity bands appear. Brookite’s crystal structure is orthorhombic, and symmetry considerations foresee 36 Raman allowed modes, many more than in the case of tetragonal rutile and anatase polymorphs [60]. Zhang et al. [61] observed 15 Raman bands in the 100–700 cm^−1^ range, with the symmetries of A_1g_ (122, 149 (most intense), 192, 244, 405, and 630 cm^−1^), B_1g_ (212, 321, and 504 cm^−1^), B_2g_ (364, 392, 463, and 583 cm^−1^), and B_3g_ (284 and 455 cm^−1^). Analysis of the C-TiProp spectrum shows that the observed new weak bands at 207, 242, 316, and 360 cm^−1^ are assignable to brookite admixture, which also contributes to the most intense band at 150 cm^−1^. 

TiO_2_ NPs prepared by the A method from the TiCl_4_ precursor yield Raman spectra showing distinct bands at 110, 247, 443, 607, and 685 cm^−1^, which, as argued above, points to the presence of nanocrystalline rutile, in agreement with the A-TiCl diffraction pattern. In addition, a low-intensity band at 150 cm^−1^, i.e., in the position corresponding to the most intense anatase and/or brookite maxima, indicates a trace admixture of either of these phases, undetected by XRD. 

The Raman spectrum of B-TiCl shows only the features of nanocrystalline rutile, as evidenced by the bands at 110, 247, 441, 606, and 680 (shoulder) cm^−1^. The most likely reason for the phase purity of B-TiCl, as opposed to A-TiCl in which rutile is contaminated with traces of anatase and/or brookite, is the higher acidity of the aqueous phase in microemulsion B, which, as argued earlier, favors the formation of rutile. 

The spectrum of C-TiCl is a mixture of features characteristic of rutile, brookite, and/or anatase, the result expected on the basis of XRD data. It is similar to that of C-TiProp, but the bands characteristic of brookite are better resolved, and, in addition to maxima at 207, 242, 316, and 360 cm^−1^, the band at 120 cm^−1^ becomes visible as well. 

Thus, vibrational spectroscopy confirms the identity of the main TiO_2_ phases present in the synthesized nanopowders. FTIR spectroscopy shows that their surface is highly hydrated/hydroxylated, with the samples prepared by method C being particularly rich in strongly hydrogen-bonded surface hydroxyls. Raman spectra mostly agree with the phase composition determined by XRD, but in the case of rutile prepared by method A from the TiCl_4_ precursor, they reveal the presence of trace amounts of anatase/brookite undetected by XRD.

### 3.5. UV–vis Diffuse Reflectance Spectroscopy

**Figure 7:**
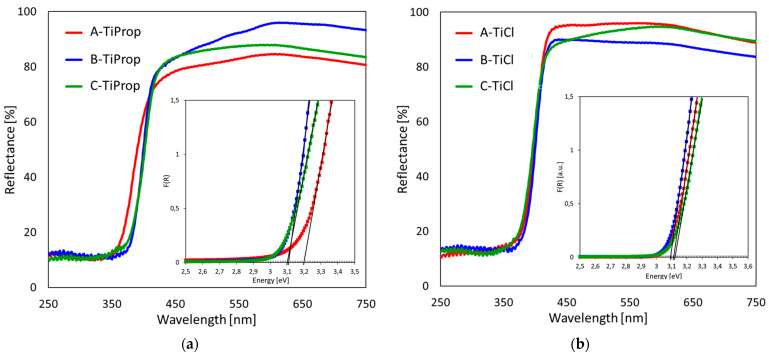
DRS spectra of: (**a**) TiO_2_ NPs prepared by method A, B, or C from the Ti isopropoxide precursor; (**b**) TiO_2_ NPs prepared by method A, B, or C from the TiCl_4_ precursor. Inserts show the plots of Kubelka–Munk function F(R) vs. energy of irradiation, used for the determination of band gap energy.

In order to obtain insight into the optical properties of the prepared titania nanopowders, the samples were subjected to UV–vis DRS analysis and the selected spectra are shown in Figure 7. The type of the alkoxide precursor used for the synthesis of TiO_2_ NPs had no meaningful impact on the spectra of materials obtained by a given method, therefore Figure 7a depicts, as an example, the spectra of samples prepared from Ti isopropoxide. Figure 7b shows the spectra recorded for TiCl_4_-derived NPs. The inserts show the plots of respective Kubelka–Munk functions F(R) used for the band gap determination by graphical extrapolation of the linear part of the F(R) plot. 

Comparison of the spectra in Figure 7a shows that the reflectance edge of the A-TiProp sample is shifted toward lower wavelengths, compared to the B-TiProp and C-TiProp plots. As a result, the Eg value of A-TiProp (3.20 eV) is higher than the band gap values found for B-TiProp (3.11 eV) and C-TiProp (3.10 eV). Similar dependencies are observed for samples prepared from other alkoxides (Table 1). Of the three TiO_2_ polymorphs, rutile has the smallest bandgap (~3.0 eV), followed by anatase (~3.2 eV), and brookite (~3.3 eV) [62]. Therefore, the higher Eg values calculated for TiO_2_ NPs obtained from alkoxides by method A, yielding anatase, compared to those determined for NPs obtained by method B, yielding rutile, follow the expected trend. Noteworthy, the band gap energy of C-TiProp, which is a mixture of rutile, anatase, and brookite, is similar to that of B-TiProp, which is pure rutile. Such a phenomenon has been previously reported and attributed to the formation of defects at the junction of different polymorphs, causing a reduction of the band gap [63].

The reflectance edges of DRS spectra recorded for samples prepared from TiCl_4_ nearly overlap. As a consequence, the Eg values are very close and equal—3.11, 3.09, and 3.12 eV for A-TiCl, B-TiCl, and C-TiCl, respectively. Similar to alkoxide-derived TiO_2_ NPs, the multiphase C-TiCl sample has a band gap very close to those determined for A-TiCl and B-TiCl, which are pure rutile materials.

### 3.6. Photocatalytic Experiments

**Figure 8:**
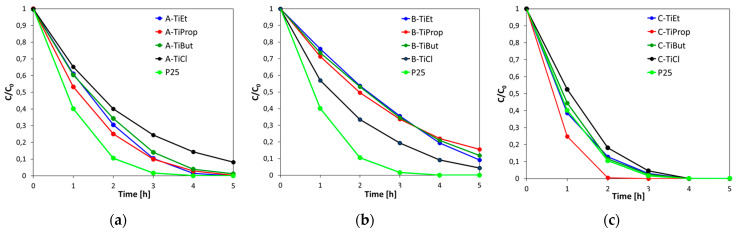
Decrease in rhodamine B concentration over the time of irradiation: (**a**) TiO_2_ NPs prepared by method A; (**b**) TiO_2_ NPs prepared by method B; (**c**) TiO_2_ NPs prepared by method C.

The photodegradation of rhodamine B was used as a model reaction to compare the photocatalytic activities of synthesized materials. The results of experiments conducted on TiO_2_ nanoparticles prepared by methods A, B, and C are shown in Figure 8. To facilitate comparison, the activity of the reference sample P25 is shown in each graph. 

Analysis of Figure 8 clearly shows that TiO_2_ photocatalysts obtained by method C are the most active in RhB degradation and perform better than materials obtained by methods A and B. Samples of the C series prepared from alkoxides are particularly efficient, as their activity is at the level, or in the case of C-TiProp better, than that of the P25 reference. Materials obtained by synthesis according to variants A and B are also photoactive. Series A samples derived from alkoxides are more active than their series B counterparts. Within a given series, there is no clear differentiation due to the nature of the alkoxide, but the RhB degradation curves for catalysts prepared with TiCl_4_ stand out in both series. In series A, the TiCl_4_-derived sample performs poorer than materials obtained from alkoxides, while the opposite is true of series B. 

Results of the physicochemical characterization presented in previous sections are of help in finding a rationale for the observed dependencies. Among the factors that are of key importance in the determination of titania photoactivity are the phase composition, crystallinity, specific surface area, or surface adsorptive properties [64]. The investigated materials differ greatly in all these aspects. Structurally, they represent anatase, rutile, or a mixture of rutile, anatase, and brookite. The size of rutile nanocrystals ranges from about 7 to 25 nm; the crystal size of anatase is ca. 6 nm, while that of brookite is ca. 8–9 nm. Large differences are also observed in the textural parameters (S_BET_ from 56 to 114 m^2^g^−1^ for NPs from TiCl_4_ precursor, and from 41 to 235 m^2^g^−1^ for NPs from alkoxide precursors). In addition, the adsorptive properties of alkoxide-derived samples of the C series differ from the rest of the catalysts, as evidenced by the presence of strongly hydrogen-bonded adsorbed water. 

The feature that clearly makes the catalysts of the C series exceptional is their multiphase composition, as they constitute mixtures of rutile, anatase, and brookite. Although the lowest band gap enables rutile to harvest UV-light over a wider range compared to other polymorphs, anatase is most frequently found to show higher photoactivity [60,62,64]. This has been attributed to a number of reasons, including longer charge-carrier lifetime, longer exciton diffusion length, higher charge-carrier mobility, and/or better surface adsorptive capacity of anatase. Nevertheless, this issue is still under debate and reports exist evidencing the higher photocatalytic activity of rutile [65]. In contrast to anatase and rutile, brookite is the least studied TiO_2_ polymorph, but in some cases high catalytic activity has been reported [62,66]. As for mixtures of titania polymorphs, it has been repeatedly reported that they can display synergism and be more efficient in photocatalytic applications than individual components [60,62,63,67,68]. The synergy has been attributed to the band alignment between heterophase components, interfacial charge transfer, and formation of boundary defects [60,63,67,68]. In fact, the P25 reference is itself a mixture of anatase and rutile [41]. In the case of photocatalysts synthesized by method C, the ratio of anatase to rutile to brookite shows variation. The C-TiCl sample contains the highest amount of rutile (63 wt.%) and the least amount of anatase (13%) (Table 1). In the more active alkoxide-derived members of the C series, anatase is the majority component, followed by rutile and brookite (Table 1). All samples of this series show appreciable specific surface areas. Moreover, as indicated by the FTIR spectra, the alkoxide-derived samples of the C series have also the most hydrated/hydroxylated surface. It is well-known that the surface hydroxyl groups play an important role in the photocatalytic activity of titania, because they react with photoholes to yield hydroxyl radicals, the most important intermediate species in the photocatalytic process [69]. Thus, it is proposed that the exceptionally high photoactivity of alkoxy samples belonging to the C series is due to their multiphase composition and associated favorable modification of the electronic structure, high specific surface area, and strongly hydrated/hydroxylated surface. It is noteworthy that the best C-TiProp catalyst has the most balanced phase composition, with a 39 wt.% of anatase and virtually equal content of rutile (30 wt.%) and brookite (31 wt.%), and the highest specific surface area (112 m^2^g^−1^) of all C series samples.

The main difference between the materials obtained from Ti alkoxides by method A and B is the formation of pure anatase in the former and pure rutile in the latter case. Moreover, the anatase samples are characterized by significantly higher specific surface areas than the rutile ones. Each of these characteristics can be considered as determining the higher photoactivity of the samples belonging to the A series. As for the photocatalysts obtained from TiCl_4_, according to XRD, both are rutile, with A-TiCl containing possible traces of anatase or brookite made visible by Raman spectroscopy. Seeking to justify this effect, the textural characteristics of the two samples should be taken into account. The specific surface area of B-TiCl is twice that of A-TiCl, consistent with the former’s better photoactivity. However, the observed increase in photocatalytic activity over the B-TiCl sample is lower than expected based on the specific surface area ratio. This can be attributed to the much narrower pores present in B-TiCl (APD = 3.6 nm compared to 19.8 nm in A-TiCl), which hinder the diffusion of reactants and partially counteract the effect of the increase in specific surface area. 

## 4. Conclusions

Titania nanoparticles with adjustable phase composition corresponding to anatase, rutile, or an anatase/rutile/brookite mixture were prepared at a mild temperature (80 °C) by an inverse microemulsion method in a ternary system of water/CTABr/1-hexanol. An aqueous/surfactant/oil phase weight ratio of 17:28:55 was used, with the only variables being the manner of preparation of the aqueous component and the nature of the Ti precursor added (Ti(IV) ethoxide, isopropoxide, butoxide or TiCl_4_). The results of the syntheses were as follows:

- Anatase NPs were formed when the alkoxides were added to a microemulsion containing pure H_2_O as the aqueous phase (method A);
- Rutile NPs crystallized when the alkoxides were added to a microemulsion containing 6 M HCl as the aqueous phase (method B), and when TiCl_4_ was added to a microemulsion containing water (method A) or 6 M HCl (method B) as the aqueous phase;
- Mixed-phase anatase/rutile/brookite NPs were obtained when the aqueous phase used to form the microemulsions contained Ti precursor (method C).

The acidity of the aqueous component of the microemulsion was considered to be the main factor affecting the phase composition of TiO_2_ NPs. Physicochemical characterization revealed that the synthesis products differed greatly not only with respect to their phase composition, but also crystallinity, textural properties, and surface adsorptive properties towards water. 

Photocatalytic tests showed that all synthesized TiO_2_ NPs were active in rhodamine B degradation, with mixed-phase materials obtained from alkoxy precursors showing the best performance, comparable to or better than the P25 reference. The exceptionally high photoactivity was attributed to the advantageous electronic effects known to appear in multiphase titania, high specific surface area, and strong surface hydration/hydroxylation. Among the single-phase materials, anatase samples showed, as expected, better photoactivity than rutile ones, the effect reinforced by their high specific surface area.

The proposed synthesis method offers a facile means for tailoring the crystal structure of TiO_2_ nanopowders while maintaining a high specific surface area, thus enabling the preparation of materials for applications requiring titania with a specific polymorph composition and well-developed textural properties.

## Figures and Tables

**Table 1:** Textural parameters from N_2_ adsorption/desorption isotherms at −196 °C (S_BET_—BET specific surface area, V_tot_—total pore volume, D_av_—average pore size, D_dom_—pore size of the dominant PSD maximum); TiO_2_ phase composition and crystallinity from XRD measurement; band gap energy E_g_ from DRS measurement.

Sample	S_BET_ [m^2^g^−1^]	V_tot_ [cm^3^g^−1^]	D_av_ [nm]	D_dom_ [nm]	Anatase Content/ Crystal Size [wt.%]/[nm]	Rutile Content/ Crystal Size [wt.%]/[nm]	Brookite Content/ Crystal Size [wt.%]/[nm]	E_g_ [eV]
A-TiCl	56	0.279	19.8	6.1	-	100/11.3	-	3.11
A-TiEt	206	0.318	6.2	6.1	100/6.5	-	-	3.18
A-TiProp	216	0.326	6.0	6.1	100/6.2	-	-	3.20
A-TiBut	235	0.324	5.5	6.1	100/6.3	-	-	3.18
B-TiCl	114	0.095	3.4	2.9	-	100/7.4	-	3.09
B-TiEt	41	0.125	12.2	5.9	-	100/8.7	-	3.09
B-TiProp	47	0.153	12.9	6.6	-	100/8.1	-	3.11
B-TiBut	42	0.128	12.2	6.1	-	100/8.2	-	3.12
C-TiCl	83	0.270	13.0	4.9	13/5.1	63/18.2	24/9.3	3.12
C-TiEt	100	0.313	12.5	4.9	43/5.9	35/23.0	22/8.4	3.10
C-TiProp	112	0.291	10.4	4.9	39/5.9	30/24.8	31/7.6	3.10
C-TiBut	99	0.347	14.0	6.1	47/5.6	29/23.0	23/7.6	3.09

## Data Availability

The data presented in this study are available on request from the authors.
